# The *Podospora anserina* lytic polysaccharide monooxygenase *Pa*LPMO9H catalyzes oxidative cleavage of diverse plant cell wall matrix glycans

**DOI:** 10.1186/s13068-017-0749-5

**Published:** 2017-03-11

**Authors:** Mathieu Fanuel, Sona Garajova, David Ropartz, Nicholas McGregor, Harry Brumer, Hélène Rogniaux, Jean-Guy Berrin

**Affiliations:** 1 Unité de Recherche Biopolymères, Interactions, Assemblages, INRA, 44316 Nantes, France; 2 Polytech Marseille, UMR1163 Biodiversité et Biotechnologie Fongiques, INRA, Aix-Marseille Université, Avenue de Luminy, 13288 Marseille, France; 3 Michael Smith Laboratories, University of British Columbia, 2185 East Mall, Vancouver, BC V6T 1Z4 Canada; 4 Department of Chemistry, University of British Columbia, 2036 Main Mall, Vancouver, BC V6T 1Z1 Canada; 5 Department of Biochemistry, University of British Columbia, 2350 Health Sciences Mall, Vancouver, BC V6T 1Z3 Canada; 6 Department of Botany, University of British Columbia, 6270 University Boulevard, Vancouver, BC V6T 1Z4 Canada

**Keywords:** AA9, LPMO, Lignocellulose, Biomass, Polysaccharides, Mass spectrometry, Biorefinery

## Abstract

**Background:**

The enzymatic conversion of plant biomass has been recently revolutionized by the discovery of lytic polysaccharide monooxygenases (LPMO) that catalyze oxidative cleavage of polysaccharides. These powerful enzymes are secreted by a large number of fungal saprotrophs and are important components of commercial enzyme cocktails used for industrial biomass conversion. Among the 33 AA9 LPMOs encoded by the genome of *Podospora anserina*, the *Pa*LPMO9H enzyme catalyzes mixed C1/C4 oxidative cleavage of cellulose and cello-oligosaccharides. Activity of *Pa*LPMO9H on several hemicelluloses has been suggested, but the regioselectivity of the cleavage remained to be determined.

**Results:**

In this study, we investigated the activity of *Pa*LPMO9H on mixed-linkage glucans, xyloglucan and glucomannan using tandem mass spectrometry and ion mobility–mass spectrometry. Structural analysis of the released products revealed that *Pa*LPMO9H catalyzes C4 oxidative cleavage of mixed-linkage glucans and mixed C1/C4 oxidative cleavage of glucomannan and xyloglucan. Gem-diols and ketones were produced at the non-reducing end, while aldonic acids were produced at the reducing extremity of the products.

**Conclusion:**

The ability of *Pa*LPMO9H to target polysaccharides, differing from cellulose by their linkages, glycosidic composition and/or presence of sidechains, could be advantageous for this coprophilous fungus when catabolizing highly variable polysaccharides and for the development of optimized enzyme cocktails in biorefineries.

**Electronic supplementary material:**

The online version of this article (doi:10.1186/s13068-017-0749-5) contains supplementary material, which is available to authorized users.

## Background

The use of plant biomass represents an attractive alternative to fossil-based technologies for the production of high-value chemicals [1]. In nature, filamentous fungi produce lignocellulose-degrading enzymes to acquire carbon from plant biomass. Different types of mechanisms for the deconstruction of plant cell walls have been described in saprotrophic fungi [2], but the involvement of oxidative enzymes was largely underestimated. The recent discovery of a new class of oxidative enzymes, namely lytic polysaccharide monooxygenases (LPMOs), has dramatically broadened the concept of the enzymatic deconstruction of plant cell wall polysaccharides [3–5]. LPMOs represent key cellulolytic enzymes that act at the surface of fibers where they mediate oxidative cleavage of polysaccharide chains. In industry, addition of LPMOs to cellulolytic cocktails leads to the reduction of the enzyme loading required for efficient saccharification of cellulosic biomass [6, 7].

Known LPMOs are grouped into the families AA9, AA10, AA11 and AA13 in the CAZy classification [8]. LPMOs all feature a similar histidine brace coordinating the copper ion responsible for the oxidative cleavage of the substrate [9]. Members of the AA9 family are mainly active on cellulose. The few characterized members from AA11 and AA13 families are active on chitin and starch, respectively [10, 11]. In contrast, the AA10s are mostly found in bacteria and exhibit activity on cellulose or chitin [12]. Beside cellulose, AA9 LPMOs are also known to act on xyloglucan and glucomannan [13] as well as soluble cellodextrins [14–16]. Activity on xylan was detected only when xylans were in complex with cellulose chains [17].

Lytic polysaccharide monooxygenases from the AA9 family are exclusively found in fungi with large expansion of genes in white-rot fungi and some ascomycetes. The coprophilous fungus *Podospora anserina* displays an impressive array of CAZymes [18, 19] with 189 glycoside hydrolases and 33 genes encoding AA9 LPMOs (*Pa*LPMO9), of which seven have been characterized biochemically [15, 20]. These LMPOs are able to oxidatively cleave cellulose with different regioselectivity. For instance, *Pa*LPMO9E was shown to produce exclusively C1-oxidized products, while *Pa*LPMO9H was reported to release both C1- and C4-oxidized products from cellulose [15, 20]. In addition, the *Pa*LPMO9H displayed relatively broad substrate specificity demonstrated by enzyme activity assays using cellulose and cello-oligosaccharides. Activity of *Pa*LPMO9H on different kinds of hemicelluloses was suggested based on hydrogen peroxide assay results. Indeed, the repression of hydrogen peroxide production in the presence of different hemicellulose substrates was significant and concentration-dependent for barley β-glucan, glucomannan, lichenan, and xyloglucan while curdlan, pectin, and xylan had no effect on hydrogen peroxide production [15]. The degradation products and regioselectivity of *Pa*LPMO9H acting on these substrates remained undetermined.

In the present work, a comprehensive oligosaccharide structural analysis was performed by tandem mass spectrometry (MS) and ion mobility–mass spectrometry on the released products of *Pa*LPMO9H following incubation with four hemicellulosic substrates: lichenan, barley mixed-linkage glucan (MLG), konjac glucomannan (GM) and tamarind xyloglucan (XyG). These polysaccharides are widely represented in the cell walls of different monocotyledonous or dicotyledonous plants, and they exhibit different structural features [21]. Similar to cellulose, all contain some (1 → 4)-linked β-d-glucose residues. Lichenan and MLG are polymers of β-d-glucosyl residues linked through (1 → 4) and (1 → 3) linkages. GM is composed of β-d-glucose and β-d-mannose residues, linked through (1 → 4) bonds, while XyG is a branched polysaccharide, with a backbone of (1 → 4)-linked β-d-glucose residues, most of which are substituted with (1 → 6)-linked α-d-xylose sidechains which are sometimes further extended with (1 → 2)-linked β-d-galactose residues. Our objective was to determine the specificity and selectivity of the *Pa*LPMO9H enzyme for these structures, differing from cellulose by their linkages, glycosidic composition and/or the presence of sidechains.

## Methods

### Chemicals, substrates and standards

HPLC-grade methanol (MeOH) was purchased from Carlo-Erba (Peypin, France). Ultrapure Water was obtained from a milli-Q apparatus (Merck Millipore, Saint Quentin en Yvelines, France). All other chemicals were purchased from Sigma-Aldrich.

Polysaccharides including lichenan from Icelandic moss, barley MLG, tamarind XyG, konjac GM and β(1,3)/β(1,4)-oligosaccharides including G4A (Glc-β(1,3)-Glc-β(1,4)-Glc-β(1,4)-Glc), G4B (Glc-β(1,4)-Glc-β(1,4)-Glc-β(1,3)-Glc) and G4C (Glc-β(1,4)-Glc-β(1,3)-Glc-β(1,4)-Glc) were purchased from Megazyme (Bray, Ireland). XXXGXXXG (XGO14) was prepared and analyzed by HPAEC-PAD as described in McGregor et al. [22]. Phosphoric acid cellulose was prepared as described in Bennati-Granier et al. [15].

### Enzyme production

*Pa*LPMO9H from *Podospora anserina* (Genbank ID CAP61476) was expressed in *Pichia pastoris*, produced in a bioreactor and purified as described previously [15].

### Cleavage assays

All the cleavage assays (300 µL liquid volume) contained 4.4 µM of *Pa*LPMO9H, 1 mM of ascorbate and 0.05% (w/v) of hemicellulose or 0.2 mM XGO14 in milli-Q water. No buffer was used to avoid the formation of multiple salt adducts during the mass spectrometry analyses causing loss of sensitivity or misinterpretations. The enzyme reactions were performed in 2-mL tubes and incubated in a thermomixer (Eppendorf) at 40 °C and 500 rpm. After 24 h of incubation, the enzymatic reaction was stopped by filtration though a 10-kDa cutoff polyether sulfone membrane and the soluble products were analyzed by mass spectrometry.

### Mass spectrometry

Experiments were performed on a Synapt G2Si high-definition mass spectrometer (Waters Corp., Manchester, UK). Two types of mass measurements were performed on the samples. First, a mass profile was collected for a mass range of 350–2000 *m/z*. Second, ion mobility (IM) was activated to collect ion mobility–mass spectra separating possible isomers and/or reducing interference from sample impurities. IM wa performed in a traveling-wave ion mobility (TWIM) cell. The gas flows were held at 180 mL min^−1^ He in the helium cell and at 90 mL min^−1^ N_2_ in the mobility cell. The IM traveling-wave height was set to 40 V and its wave velocity was set to 450 m s^−1^ for positive ionization mode and 550 m s^−1^ for negative ionization mode. Ions of interest were fragmented after the gas-phase separation by collision-induced dissociation in the transfer cell of the instrument, with an adjustment of the collision energies such that a significant fragmentation was obtained while keeping an observable proportion of the intact precursor. Samples were diluted tenfold in MeOH/H_2_O (1:1, v/v) and infused at a flow rate of 5 μL min^−1^ in the instrument. The instrument was operated in positive or negative polarity and in “sensitivity” mode. Data acquisition was carried out using MassLynx software (V4.1).

## Results and discussion

Different sources of hemicelluloses were incubated with *Pa*LPMO9H under ascorbate conditions and their reaction products were characterized by mass spectrometry. Four substrates were considered: lichenan, MLG, XyG and GM. Cellulose was also included in the study as a reference substrate. The activity of *Pa*LPMO9H products on cellulose has been described in detail and was shown to generate singly and doubly oxidized products at the C1 and/or C4 positions. This results in the formation of gem-diols/ketones at the newly formed “non-reducing” end or aldonic acids at the newly formed “reducing” end [15].

### PaLPMO9H demonstrates broad specificity toward β-(1 → 4)-linked glucans

**Fig. 1:**
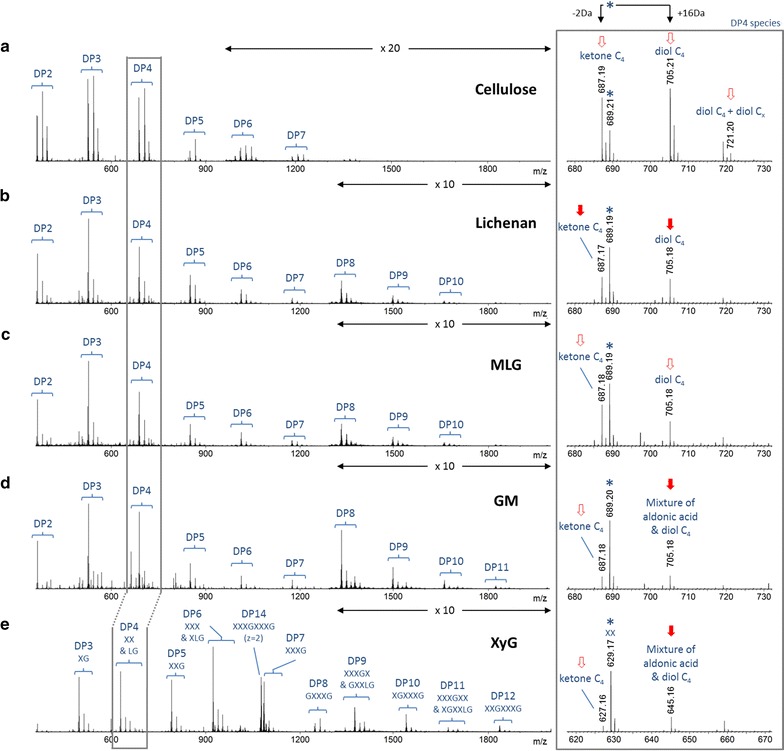
ESI-MS spectra showing the degradation products generated from several cellulosic and hemicellulosic substrates by *Pa*LPMO9H under ascorbate conditions. **a** Cellulose; **b** lichenan; **c** MLG; **d** GM; **e** XyG. Species were detected as sodium adducts ([M+Na]^+^) except for XXXGXXXG which was detected as [M+2Na]^2+^. The main degrees of polymerization (DP) were annotated. *Right panel* enlarged view of the DP4 species, for all substrates; *stars* the non-modified product. Peaks indicated by an *arrow* were attributed to the oxidized species (−2, +16, +32 Da relative to the non-modified species) and were further characterized by tandem MS (*full arrow* data presented in the following sections; *empty arrows* data not shown). Labels of the oxidized species were proposed based on tandem MS results. For cellulose: “diol C4+ diol C*x*” indicates a double oxidation (diols): one has been localized at C4 of the non-reducing end, while the second one could not be precisely localized and is either localized at the reducing end or non-reducing end

As shown by the mass spectra in Fig. 1b–e, the incubation of *Pa*LPMO9H with the four hemicellulosic substrates produced some oligosaccharides having degrees of polymerization (DPs) ranging from 2 to 12, confirming the depolymerizing activity of the enzyme on all of these structures. The most abundant peaks ranged between DP2 and DP4 for lichenan, MLG, and GM. In the case of XyG, DP6 and DP7 were detected with the highest intensity. In addition, the presence of some species with a mass shift of −2 (ketone or lactone) and +16 (gem-diol or aldonic acid) Da relative to the non-modified oligosaccharide suggested an oxidative cleavage activity, in agreement with the previous observations on cellulose [15]. The profiles of the reaction products were quite similar for all hemicellulosic substrates (Fig. 1, right panel). The non-modified species were always the most intense, regardless of the DP, while the intensity of the −2 Da species was either higher or similar to that of +16 Da species. The situation was slightly different when *Pa*LPMO9H was incubated with cellulose (Fig. 1a). The species at −2 and +16 Da were the most intense ions. One additional species of low intensity was detected with a mass shift of +32 Da (*m/z* 721.20) from the related non-modified DP. This species has been described as a doubly oxidized product, with one gem-diol on the non-reducing end (C4), and another oxidation either at the reducing end or at the non-reducing end [15].

These results unambiguously confirmed that *Pa*LPMO9H enzyme exhibits a broad specificity toward hemicelluloses compared to other AA9 LPMOs. Subtle differences were observed with respect to the action on cellulose, among which the apparent absence of double-oxidation. In-depth characterization of the reaction products was further carried out by tandem MS to explore the regioselectivity of the oxidative cleavage.

### PaLPMO9H oxidatively cleaves β-(1 → 4)- and β-(1 → 4; 1 → 3)-linked glucosidic substrates

**Fig. 2:**
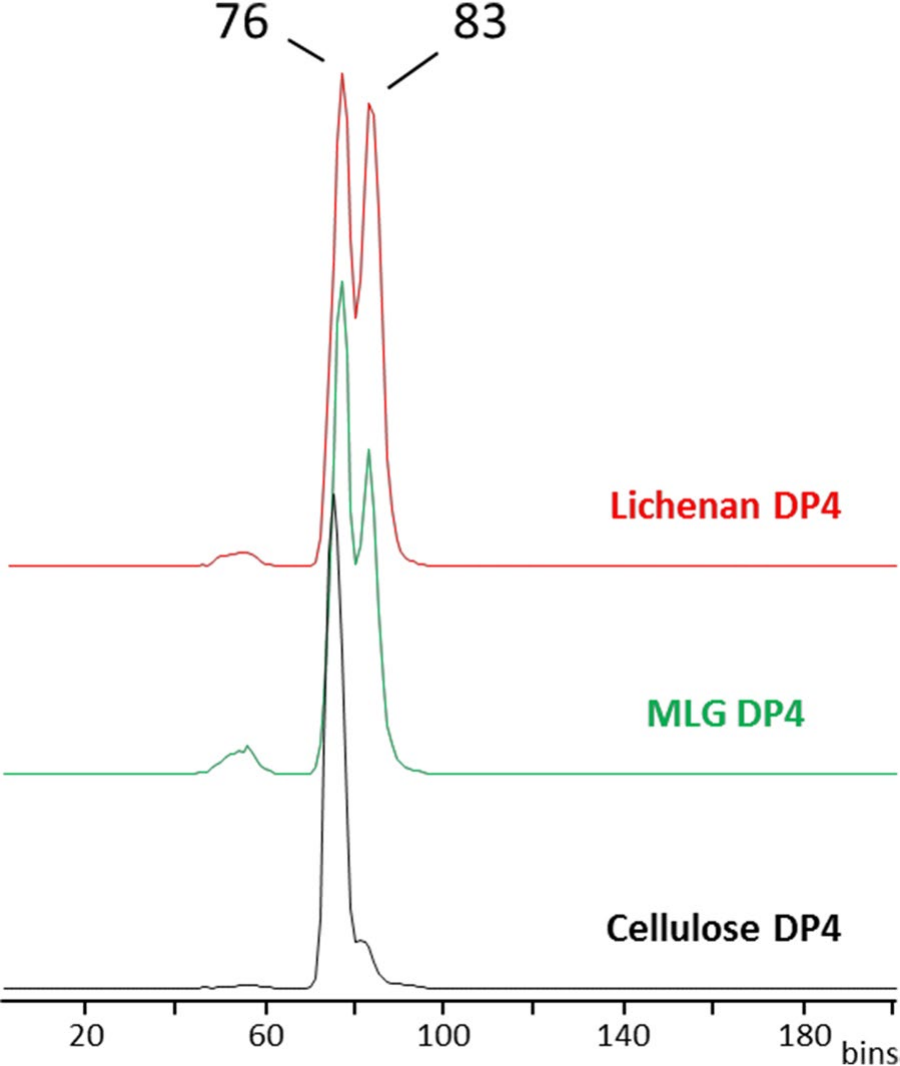
Ion mobility recorded in positive ionization mode for the non-modified DP4 species (*m/z* 689.2 Da) released from the incubation of *Pa*LPMO9H with cellulose (*black trace*), MLG (*green trace*), and lichenan (*red trace*). Drift times are indicated in bins (arbitrary units). The shoulder peak in the case of cellulose DP4 was attributed to a slight heterogeneity in the gas-phase isoforms

Lichenan and MLG are each composed of a linear chain of β-d-glucosyl residues linked by (1 → 4) and (1 → 3) linkages. The proportion and distribution of these β-(1 → 4) and β-(1 → 3) linkages vary between these two substrates; the average ratio of β-(1 → 3):β-(1 → 4) bonds is roughly 1:3 and 1:2 for MLG and lichenan, respectively. To differentiate possible linkage isomers among the reaction products, ion mobility was used in combination with tandem MS. Ion mobility separates species based on their gas-phase conformation prior to their mass measurement. Figure 2 shows the mobilograms recorded for the non-modified species of DP4 obtained for lichenan, MLG and cellulose. As cellulose is exclusively composed of β-(1 → 4) linkages, its degradation was expected to produce a single DP4 species that could be used as a reference. Figure 2 shows that a major peak at a drift time (dt) of 76 bins was detected for the natriated non-oxidized DP4 ion (*m/z* 689.2) arising from cellulose. In contrast, mobility traces indicated the clear presence of at least two gas-phase isoforms for the DP4 released from lichenan and MLG. One of those (dt = 76 bins) corresponded to a DP4 species containing three consecutive β-(1 → 4) linkages (cellotetraose). The second one (dt = 83 bins) was assumed to contain both β-(1 → 3) and β-(1 → 4) linkages. This interpretation was supported by the comparison with three DP4 commercial standards, all containing one β-(1 → 3) linkage (Additional file 1: Figure S1). The presence of one β-(1 → 3) linkage in the oligosaccharide changes the shape of the gas-phase ions and increases the drift time compared to the structure with three consecutive β-(1 → 4) linkages. Similar profiles of ion mobility were observed for the ions having a mass shift of −2 and +16 Da (*m/z* 687.2 and 705.2, respectively) from the non-modified DP4, which likely correspond to oxidized products (Additional file 1: Figure S2).

The results thus showed that DP4 species released from lichenan and MLG by *Pa*LPMO9H had two main types of structures: one displaying three consecutive β-(1 → 4) linkages, the other encompassing at least one β-(1 → 3) linkage. MLG and lichenan contain a significant proportion of β-(1 → 3) linkages. On average, the ratio of (1 → 3) to (1 → 4) β-linkages is of 1:3 to 1:2 in these two polymers. In barley MLG, cellodextrin units of two (DP3) or three (DP4) adjacent β-(1 → 4) linkages are predominant, while longer cellodextrin sequences contributed to less than 10% of the total polysaccharide chain in the starchy endosperm [23]. If one assumes that DP4 structures of different shapes have comparable ionization efficiency in MS, the peak area in Fig. 2 gives an estimate of the proportion of the two populations of DP4 differentiated by their ion mobility. The DP4 made of three consecutive β-(1 → 4) bonds was released in high proportion following action of *Pa*LPMO9H on MLG and lichenan substrates. DP5 species were also released from MLG and lichenan (Additional file 1: Figure S3). The most abundant peak (dt = 92 bins) falls at the same drift time as the one measured from cellulose. Without excluding that those species arise in part from long cellodextrin stretches in the polymer, their abundance suggests that β-(1 → 3) bonds linking consecutive cellotetraosyl units were cleaved by *Pa*LPMO9H. Thus, in addition to β-(1 → 4) linkages, *Pa*LPMO9H appears able to catalyze the oxidative cleavage of β-(1 → 3) linkages in β-(1 → 4; 1 → 3)-linked glucosidic substrates (MLG and lichenan).

**Fig. 3:**
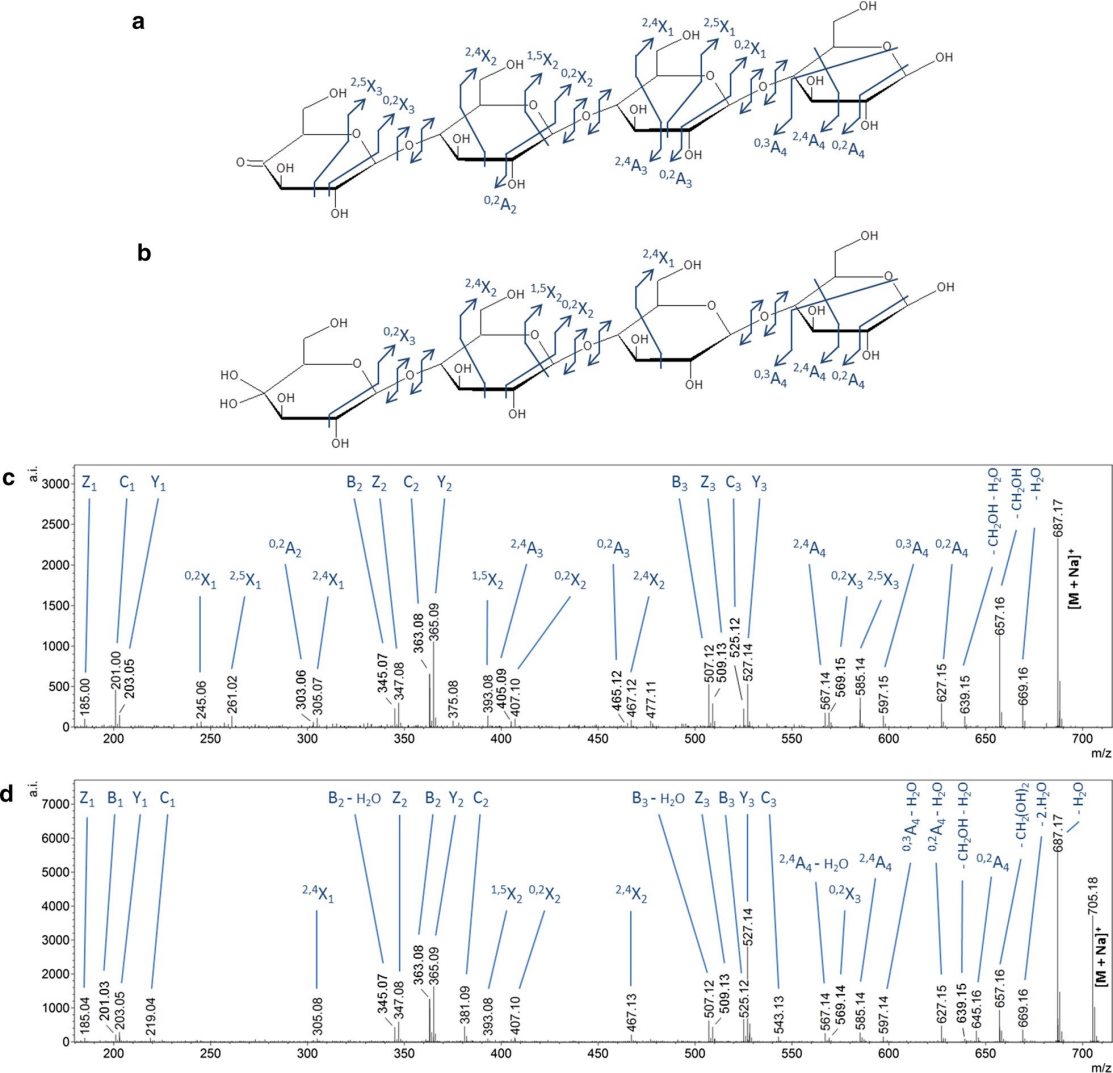
Tandem MS spectra of the oxidized species (ketone and gem-diol, detected at *m/z* 687.17 and 705.18, respectively) generated from lichenan by *Pa*LPMO9H under ascorbate conditions. These spectra represent the fragmentation of the isoforms with a drift time of 76 bins in ion mobility. These isoforms were attributed to structures composed of only (1→4)-linked β-d-glucose residues. **c** Tandem MS spectrum of the ketone form; **d** tandem MS spectrum of the gem-diol form. Observed fragments are depicted on the structures in **a** and **b** for spectra **c** and **d**, respectively

The two DP4 oxidized degradation products (−2 and +16 Da compared to the non-modified form) from lichenan and MLG were further fragmented to confirm their structures. In each case, ion mobility was used to distinguish between the isomers arising from β-(1 → 4) and/or β-(1 → 3) linkages. In accordance with the above discussion, the isoform displaying the lower drift time for each of these ions was attributed to a β-(1 → 4)-linked oligosaccharide while the second isoform (higher drift time) presumably contained a mixture of β-(1 → 3) and β-(1 → 4) bonds. The corresponding tandem MS spectra are displayed in Fig. 3. Fragmentation of the ion at *m/z* 687.17 (−2 Da compared to the non-modified DP4, Fig. 3c) led to several fragments that are characteristic of a ketone form at the non-reducing end (^2,5^X_1_, ^1,5^X_2_, ^2,5^X_3_). In contrast, the species at *m/z* 705.18 (+16 Da compared to the non-modified DP4, Fig. 3d) exhibited a double loss of water from the parent ion (indicated as −2 H_2_O on the fragmentation spectrum) under fragmentation, which is a signature of an oxidized non-reducing end in the gem-diol form, as reported by Isaksen et al. [14]. The fragment ^1,5^X_2_, observed at *m/z* 393.08, further confirmed this structure. Since lichenan are linked through β-(1 → 4) and/or β-(1 → 3) bonds and taking into account the observations we have made previously, the oxidative cleavage by *Pa*LPMO9H may have occurred on position C4 or C3. However, the fragmentation spectra for the two oxidized species (−2 and +16 Da) did not allow the determination of which position was oxidized among the C4 and C3 of the non-reducing end glycoside residue. Yet, for a simplified representation, the oxidation was positioned at C4 in the structures depicted in Fig. 3a, b. The second isoform separated by IM with a higher drift time was also fragmented for the two oxidized species. Similar to the first isoform, fragments indicating a ketone and a gem-diol on the non-reducing end were observed for the parent ions at *m/z* 687.17 and *m/z* 705.18, respectively (data not shown). Similar to the reaction products from lichenan, the two isoforms of the species at *m/z* 687.19 produced from the MLG substrate were found to arise from a ketone at the non-reducing end, while both isoforms of the species at *m/z* 705.18 were found to correspond to a gem-diol at the non-reducing end. In summary, *Pa*LPMO9H oxidatively cleaved lichenan and MLG substrates, producing both ketone and gem-diols species at the non-reducing end glycoside residue. The exact positioning of the oxidation (C4 or C3) remained undetermined.

### PaLPMO9H is active toward β-(1 → 4)-linked glucosidic substrates with branched sidechains

To examine the mode of action of *Pa*LPMO9H on xyloglucan, the tetradecasaccharide XXXGXXXG was used as a model substrate. Named according to the standard linear nomenclature, in which “G” represents an unbranched β-(1 → 4)-linked glucosyl residue and “X” represents β-(1 → 4)-linked Glc bearing an α-(1 → 6)-linked xylosyl branch [24], XXXGXXXG presents a highly decorated backbone consisting of eight glucosyl residues. The MS spectrum of the products of cleavage of the XXXGXXXG preparation by PaLPMO9H (Fig. 1e) showed the presence of minor peaks, whose masses indicated the presence of sidechain galactosylation. They likely arose from a DP15 XyG, which was present as minor impurity (roughly 5%) of the XXXGXXXG preparation as confirmed by both MS and HPAEC-PAD chromatogram of the substrate before incubation with *Pa*LPMO9H (Additional file 1: Figure S4). In the latter structure (likely XXLGXXXG and/or XXXGXXLG), one of the laterally branched xylose bears a β-(1 → 2)-linked galactosyl (represented as “L” [24]).

**Fig. 4:**
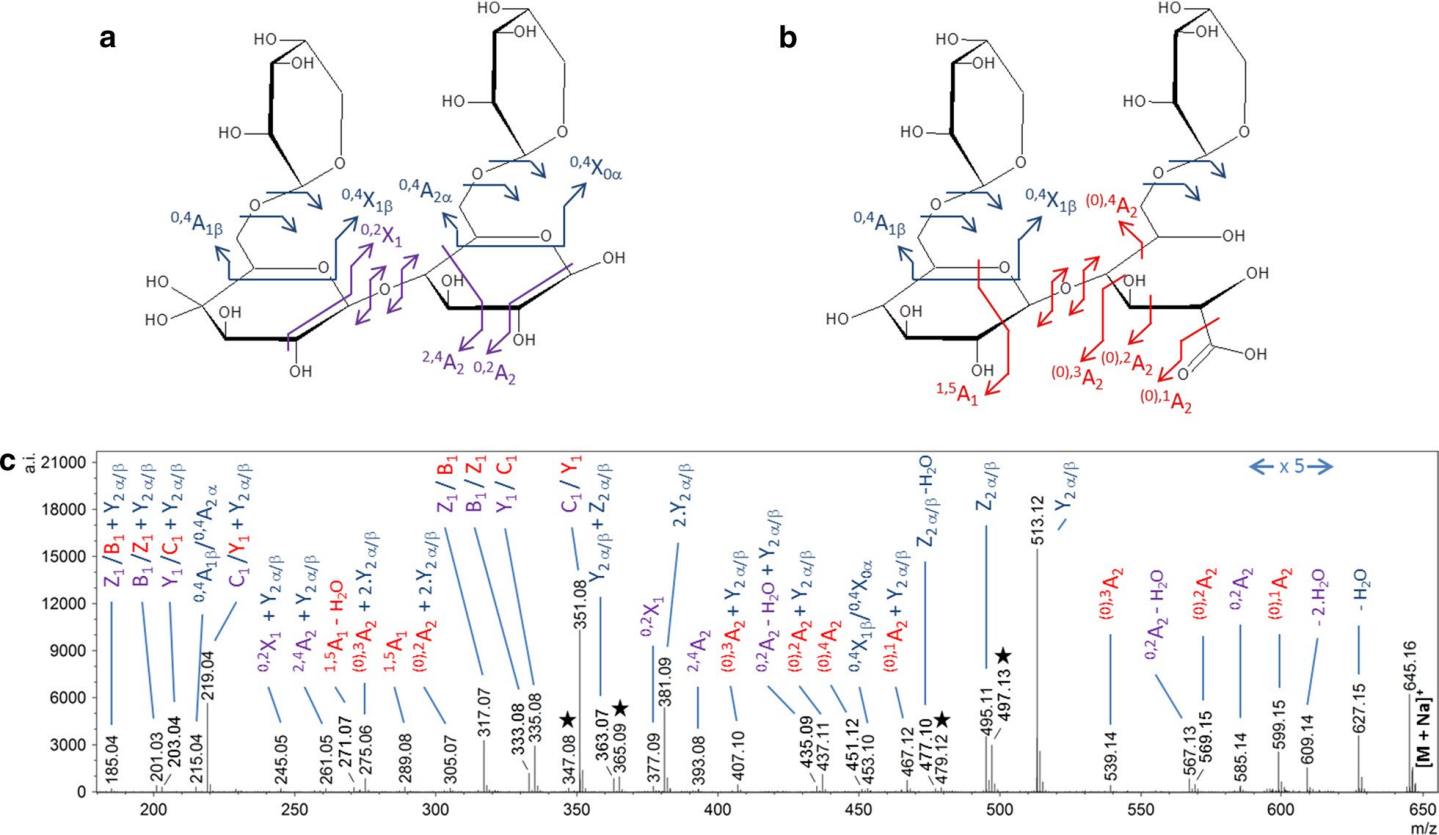
Tandem MS spectrum of the species displaying a +16 Da mass shift from the unmodified DP4 produced by *Pa*LPMO9H from XXXGXXXG (detected at *m/z* 645.16). The observed fragments indicate the co-existence of two oxidized forms: a gem-diol at the non-reducing end and an aldonic acid at the reducing end. Observed fragments are depicted on the structures in **a** and **b**. *Red fragments* are specific of the aldonic acid, *purple fragments* are specific of the gem-diol and *blue fragments* are common to both oxidized species. Fragments labeled with a *star* could not be attributed to either expected structures. Their masses match with an oxidation at one laterally branched xylose residue, which origin remains undetermined

Tandem MS analyses were performed on the species of *m/z* 645.16, which uniquely matches a hydrated oxidized XX species (+16 Da from the non-modified oligosaccharide, Fig. 1e). The fragmentation spectrum suggested the coexistence of at least two oxidized species (Fig. 4). First, the double loss of water (indicated as −2.H_2_O on the fragmentation spectrum) from the precursor ion was found, which indicated a gem-diol at the non-reducing end. The ^0,1^A_2_ fragment was also observed. This fragment ion, corresponding to a loss of 46 Da from the parent, was reported by Isaksen et al. [14] as characteristic of an acidic function at the C1 of the reducing end.

These results clearly indicated that *Pa*LPMO9H was able to oxidatively cleave β-(1 → 4)-linked glucose chains even in the presence of extensive lateral branching.

### Activity of PaLPMO9H on β-(1 → 4)-linked hetero-polysaccharides

Glucomannans are mainly straight-chain polymers of β-(1 → 4)-linked d-mannose and d-glucose. The product released at 705.18 *m/z* corresponding to a +16 Da mass shift from the non-modified DP4 was fragmented in tandem MS. The observed fragment ions showed the coexistence of two oxidized species with (i) the loss of 46 Da from the parent ion indicating a C1 oxidation (aldonic acid) at the reducing end, and (ii) a double loss of water from the precursor ion indicating a gem-diol at the non-reducing end (Additional file 1: Figure S5). On the other hand, fragmentation of the −2 Da species showed unambiguously a single species, corresponding to a ketone form at the non-reducing end. It can thus be concluded that *Pa*LPMO9H oxidatively cleaved GM by forming oxidized species both at the reducing end and the non-reducing end. Since the main chain of GM consists of β-(1 → 4)-linked residues, it can be presumed that the oxidized positions are at C1 and C4, respectively.

## Conclusion

**Fig. 5:**
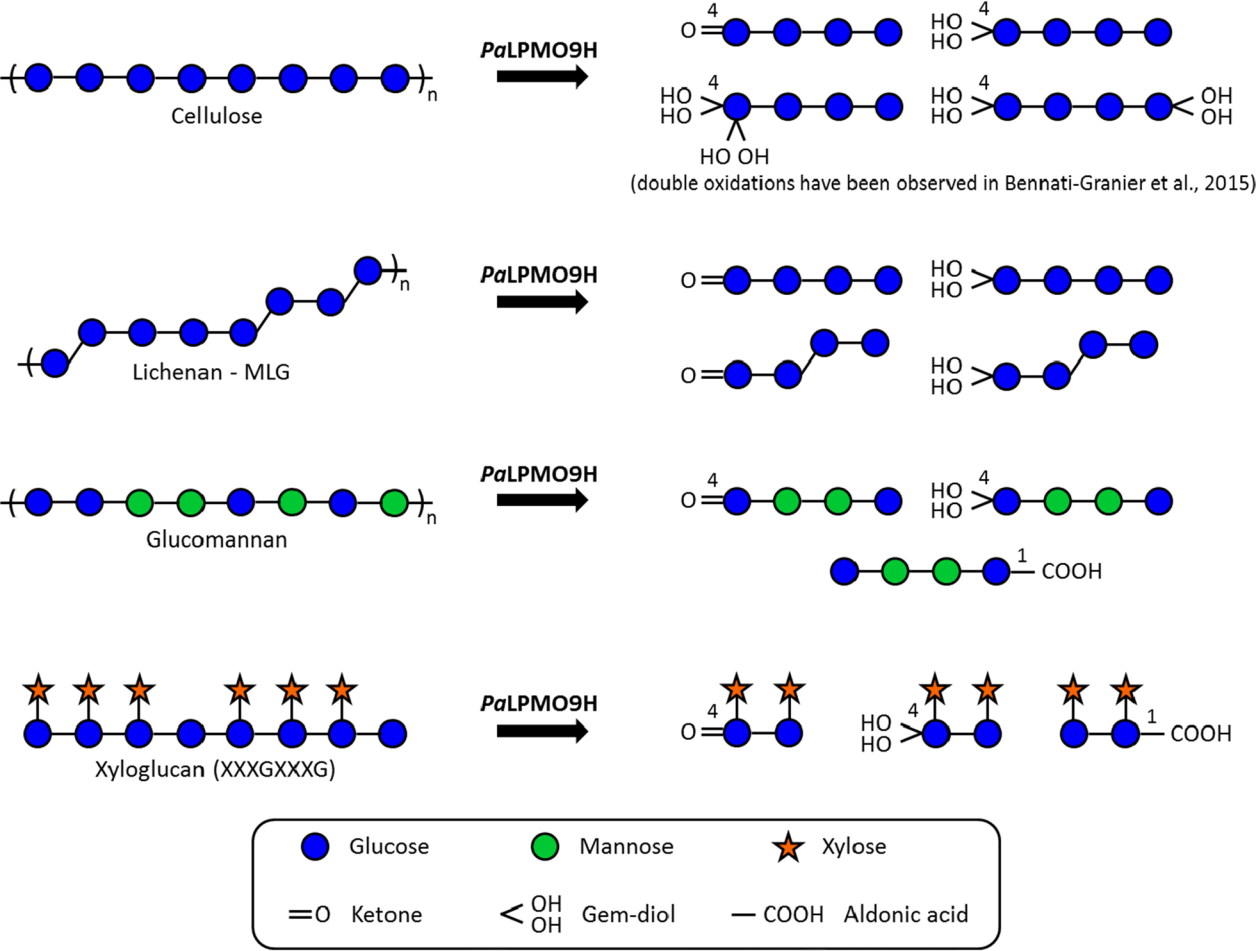
Schematic display of the broad substrate specificity of *Pa*LPMO9H according to the symbol nomenclature described in [28]. Oxidation is represented at specific carbon position (indicated by a *number*) when this position has been precisely determined by tandem MS. The chemical forms of oxidation that were characterized in this study are displayed (i.e. diol and aldonic acid for the +16 Da species, ketone for the −2 Da species). No lactone is represented, as we exclusively observed ketones for the −2 Da species. In the case of MLG and lichenan, oxidation at the non-reducing end may be localized at C3 or C4, but this was not determined. One example was arbitrarily chosen to represent the possible structures with internal β-(1→3) bonds: this bond might actually be at any backbone position

A schematic representation compiling results of the present study is proposed in Fig. 5. Altogether, the results show that *Pa*LPMO9H is able to target polysaccharides differing from cellulose by their linkage types, glycosidic composition or the presence of sidechains. The broad substrate specificity of *Pa*LPMO9H could be advantageous for *P. anserina*, which uses the most recalcitrant fraction of lignocellulose and therefore has to deal with highly variable polysaccharide structures. Recently, other fungal AA9 LPMOs have been shown to catalyze oxidative cleavage of cellulose and xyloglucan. They originate from the ascomycetes *Asperillus niger* (AN3046) [25] and *Fusarium graminearum* (*Fg*LPMO9A) [26] and the brown-rot fungus *Gloeophyllum trabeum* (GtLPMO9A-2) [27]. Compared to these enzymes, *Pa*LPMO9H seems to behave differently. Although *Fg*LPMO9A cleaves at both the C1 and C4 positions of cellulose and xyloglucan, it was not able to act on glucomannan or mixed-linkage β-glucans. Moreover, *Nc*LPMO9C that displays similar substrate specificity to *Pa*LPMO9H releases only C4-oxidized products. These subtle differences in terms of substrate specificity and regioselectivity within AA9 LPMOs could somewhat explain the multiplicity of AA9 genes in fungal saprotrophs.

## Additional file

**Additional file 1.** Structural characterization of products from incubation with the *Podospora anserina* lytic polysaccharide monooxygenase *Pa*LPMO9H on beta-1,4-linked polysaccharides. **Figure S1.** Ion mobility recorded in negative ionization mode for the non-modified DP4 species (*m/z* 665.2 Da) released from the incubation of *Pa*LPMO9H with cellulose, MLG, Lichenan and for three commercial DP4. Drift times are indicated in bins (arbitrary units). Measurements were conducted in negative ion mode (i.e. on the [M–H]^−^ species) in order to make sure that none of the gas-phase isoform arose from different sites of cationization on the oligosaccharide. Thus, the drift times cannot be directly compared to those measured in Fig. 2, neither the relative proportion of the two main populations of DP4 differentiated by their ion mobility for MLG and Lichenan. **Figure S2.** Ion mobility recorded in positive ionization mode for the oxidized DP4 species released from the incubation of *Pa*LPMO9H with cellulose, MLG, Lichenan. A:Ketone species (*m/z* 687.2 Da) B: Gem-diol species (*m/z* 705.2 Da). Drift times are indicated in bins (arbitrary units). **Figure S3.** Ion mobility recorded in positive ionization mode for the non-modified DP5 species (*m/z* 851.2 Da) released from the incubation of PaLPMO9H with cellulose (black trace), MLG (green trace), Lichenan (red trace). Drift times are indicated in bins (arbitrary units). **Figure S4.** Analysis of the tetradecasaccharide XXXGXXXG substrate, prior incubation with the *Pa*LPMO9H. A. ESI–MS spectrum showing the minor presence of XXLGXXXG or XXXGXXLG species. B. High-performance anion-exchange chromatogram indicating the presence of minor peaks likely corresponding to XXLGXXXG and XXXGXXLG. Peak integration reveals that they represent ca. 5% of the total oligosaccharide content. **Figure S5.** Tandem MS spectrum of the species displaying a +16 Da mass shift from the unmodified DP4 produced by *Pa*LPMO9H from GM (detected at *m/z* 705.2). The observed fragments indicate the co-existence of two oxidized forms: A gem-diol at the non-reducing end and an aldonic acid at the reducing end. Observed fragments are depicted on the structures in panels A and B. Red fragments are specific to the aldonic acid, purple fragments are specific to the gem-diol and blue fragments are common to both oxidized species.

## Abbreviations

AA: auxiliary activity enzyme
*Pa*LPMO9: *Podospora anserina* AA9 LPMO
LPMO: lytic polysaccharide monooxygenase
MLG: barley mixed-linkage β-glucan
XyG: tamarind xyloglucan
GM: konjac glucomannan
DP: degree of polymerization
