# Supplementary material for: The Podospora anserina lytic polysaccharide monooxygenase PaLPMO9H catalyzes oxidative cleavage of diverse plant cell wall matrix glycans
Source: Biotechnol Biofuels. 2017 Mar 11;10:63. doi: 10.1186/s13068-017-0749-5 (PMC5346257; doi:10.1186/s13068-017-0749-5)

**Supplementary data**

Structural characterization of products from incubation with the *Podospora anserina* lytic polysaccharide monooxygenase *Pa*LPMO9H on beta-1,4-linked polysaccharides

Mathieu Fanuel^1^, Sona Garajova^2^, David Ropartz^1^, Nicholas McGregor^3^, Harry Brumer^3^, Hélène Rogniaux^1,*^, Jean-Guy Berrin^2,*^

Figure S1. Ion mobility recorded in negative ionization mode for the non-modified DP4 species (*m/z* 665.2 Da) released from the incubation of *Pa*LPMO9H with cellulose, MLG, Lichenan and for three commercial DP4. Drift times are indicated in bins (arbitrary units). Measurements were conducted in negative ion mode (i.e. on the [M-H]^-^ species) in order to make sure that none of the gas-phase isoform arose from different sites of cationization on the oligosaccharide. Thus, the drift times cannot be directly compared to those measured in Figure 2, neither the relative proportion of the two main populations of DP4 differentiated by their ion mobility for MLG and Lichenan.


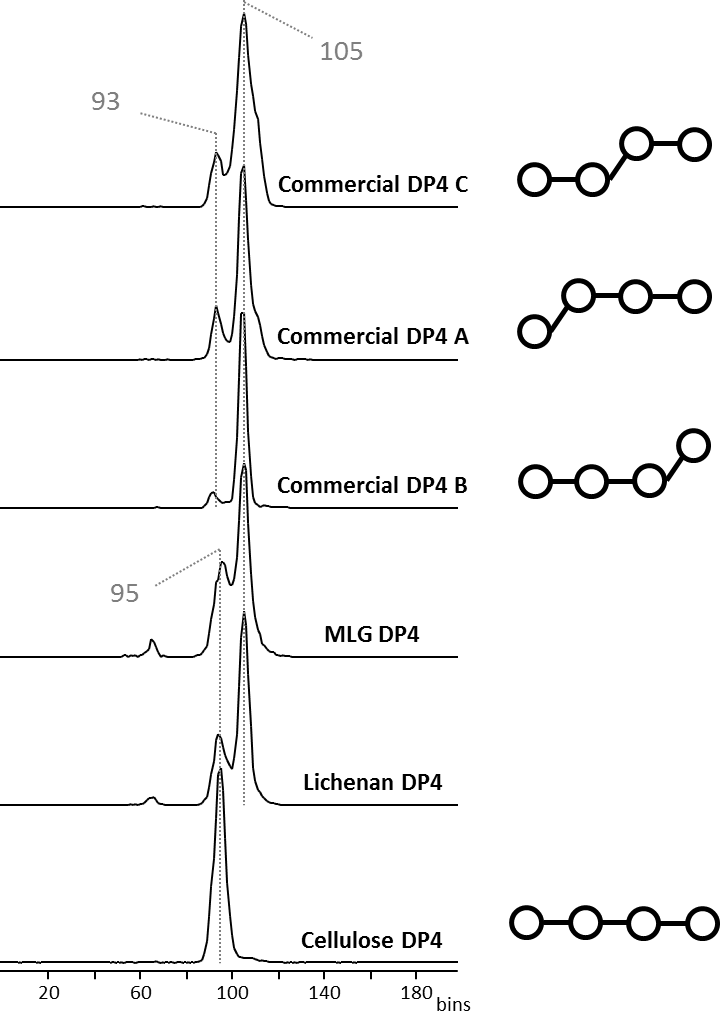


Figure S2. Ion mobility recorded in positive ionization mode for the oxidized DP4 species released from the incubation of *Pa*LPMO9H with cellulose, MLG, Lichenan. A:Ketone species (*m/z* 687.2 Da) B: Gem-diol species (*m/z* 705.2 Da). Drift times are indicated in bins (arbitrary units).


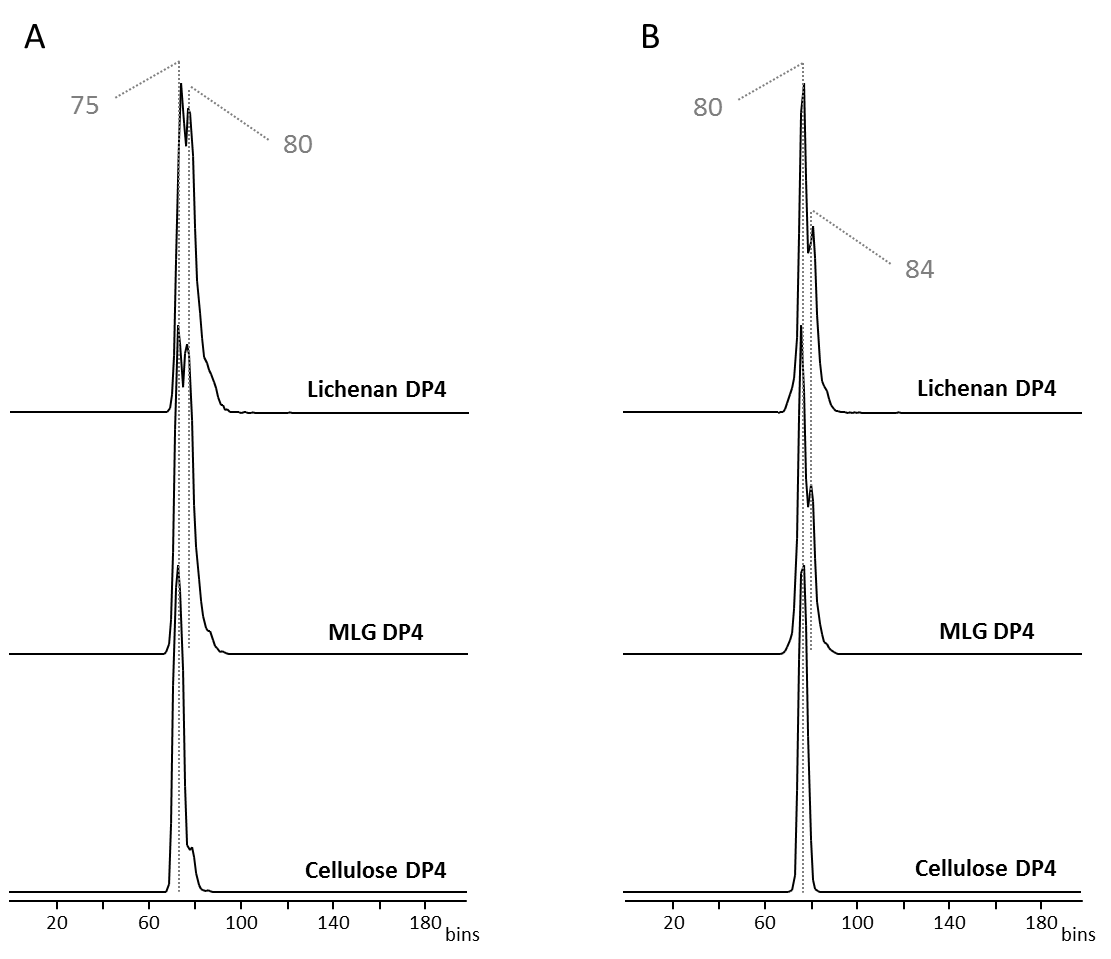


Figure S3. Ion mobility recorded in positive ionization mode for the non-modified DP5 species (m/z 851.2 Da) released from the incubation of PaLPMO9H with cellulose (black trace), MLG (green trace), lichenan (red trace). Drift times are indicated in bins (arbitrary units).


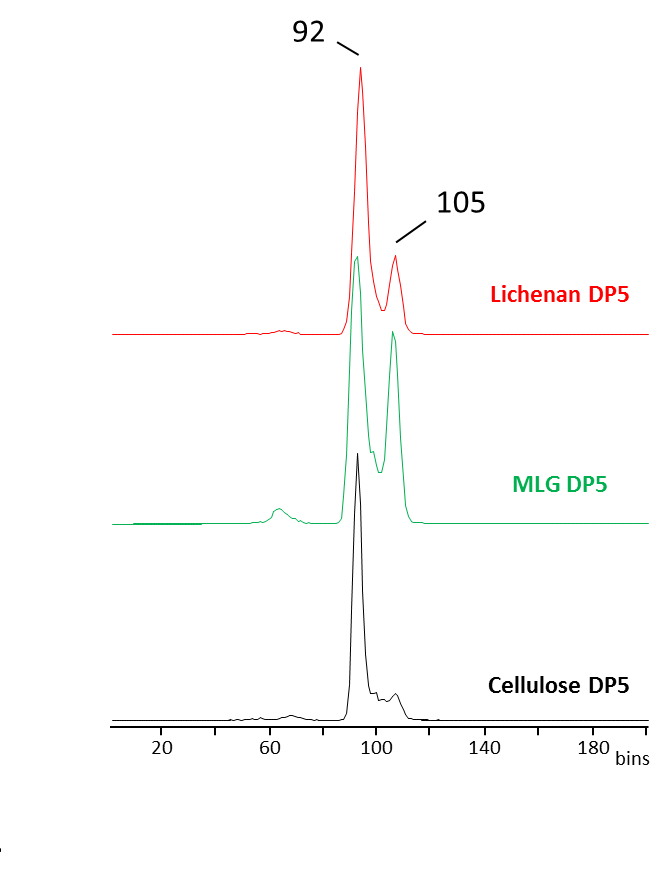


Figure S4. Analysis of the tetradecasaccharide XXXGXXXG substrate, prior incubation with the *Pa*LPMO9H. A. ESI-MS spectrum showing the minor presence of XXLGXXXG or XXXGXXLG species. B. High-performance anion-exchange chromatogram indicating the presence of minor peaks likely corresponding to XXLGXXXG and XXXGXXLG. Peak integration reveals that they represent ca. 5% of the total oligosaccharide content.

A


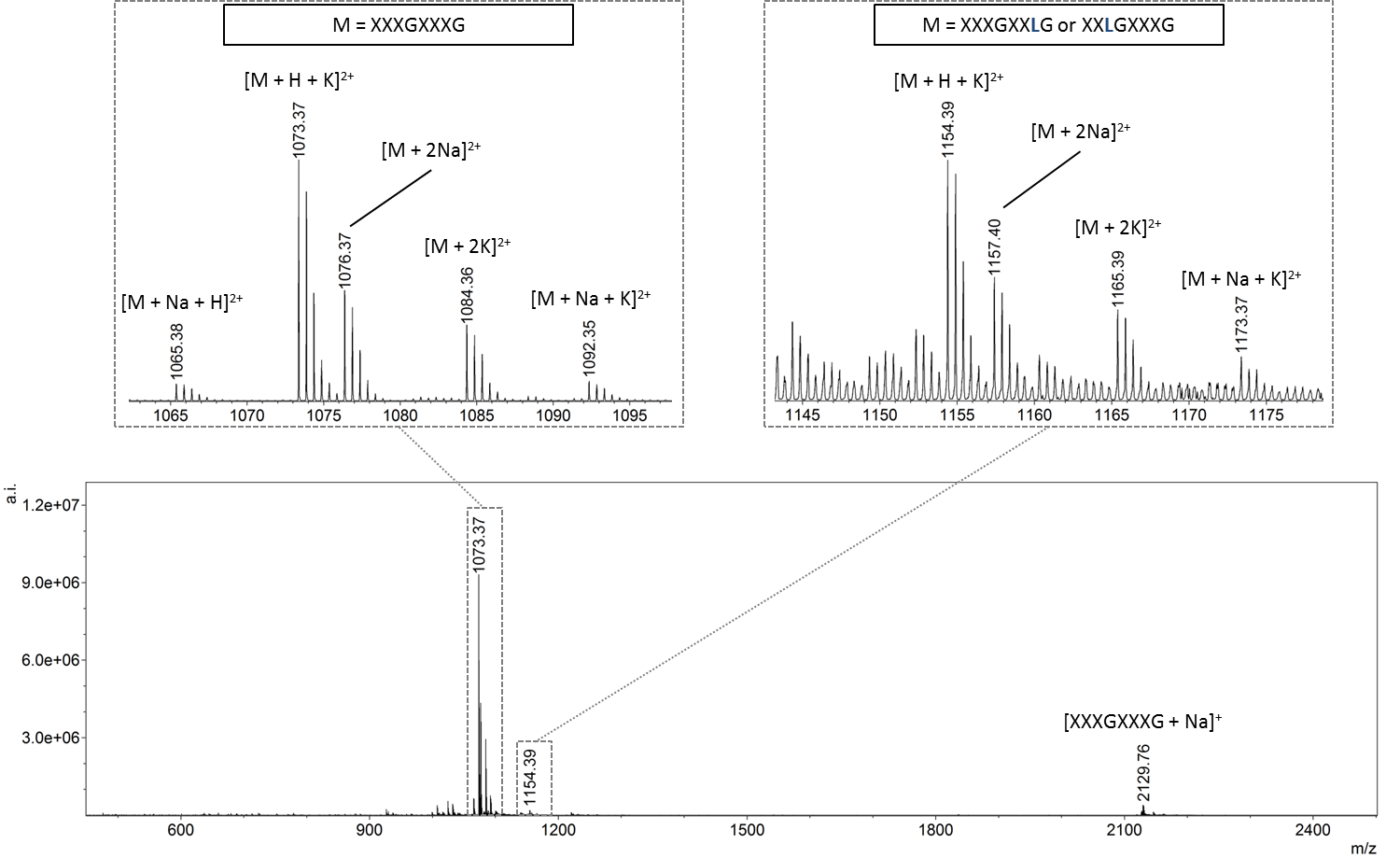


B


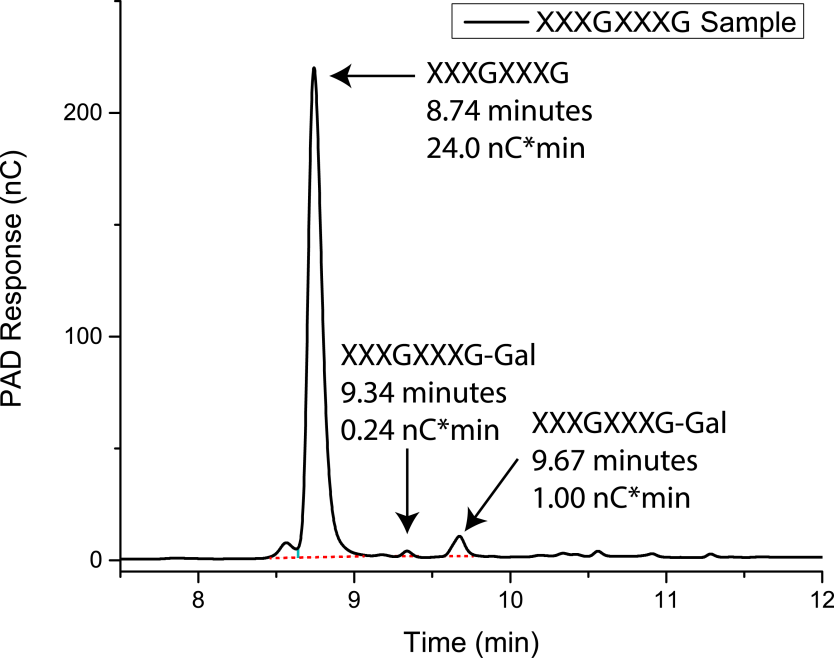


Figure S5. Tandem MS spectrum of the species displaying a +16Da mass shift from the unmodified DP4 produced by *Pa*LPMO9H from Glucomannan (detected at *m/z* 705.2). The observed fragments indicate the co-existence of two oxidized forms: A gem-diol at the non-reducing end and an aldonic acid at the reducing end. Observed fragments are depicted on the structures in panels A and B. Red fragments are specific of the aldonic acid, purple fragments are specific of the gem-diol and blue fragments are common to both oxidized species.


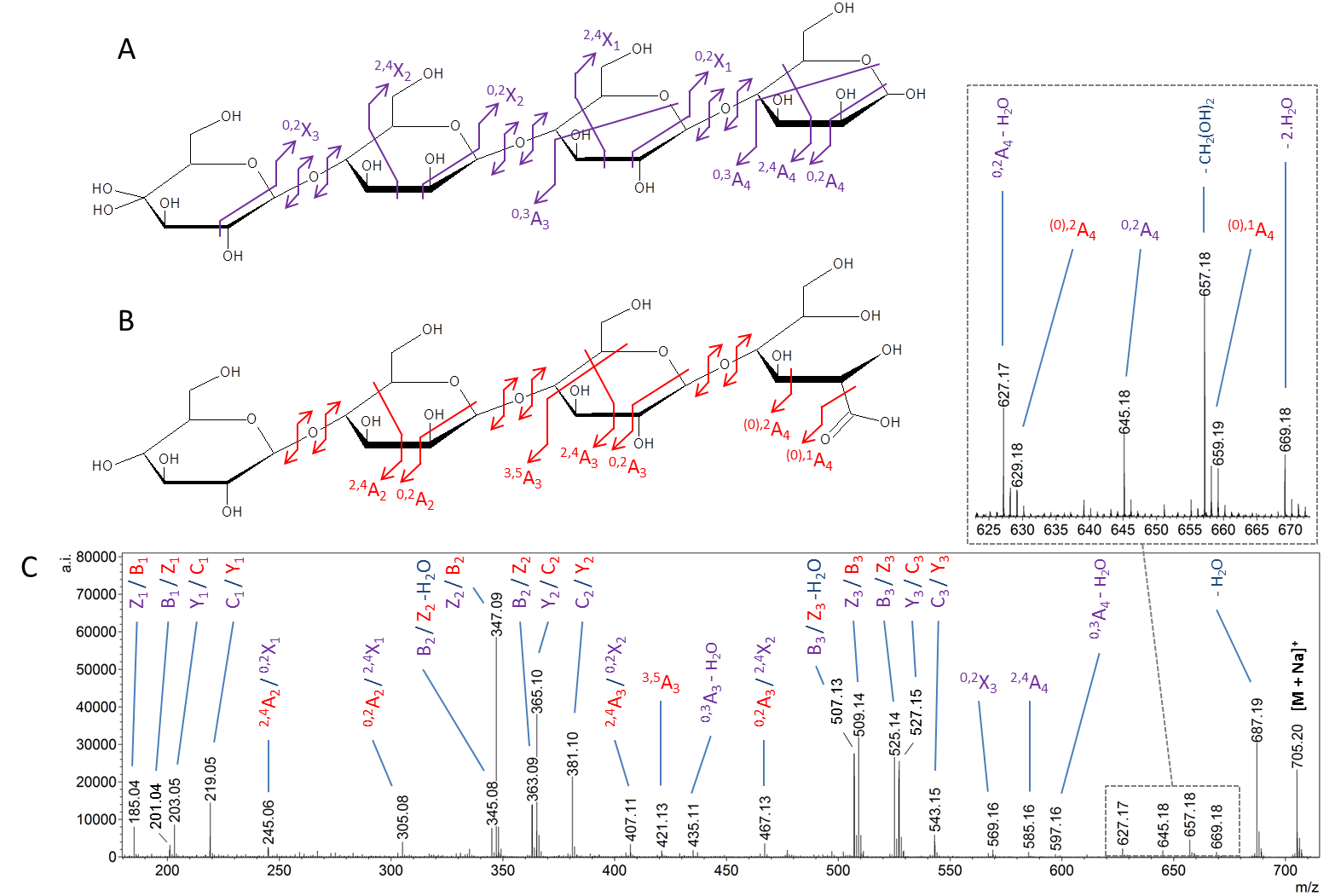

Supplement: Supplementary file 1 — Additional file 1. Structural characterization of products from incubation with the Podospora anserina lytic polysaccharide monooxygenase PaLPMO9H on beta-1,4-linked polysaccharides. Figure S1. Ion mobility recorded in negative ionization mode for the non-modified DP4 species (m/z 665.2 Da) released from the incubation of PaLPMO9H with cellulose, MLG, Lichenan and for three commercial DP4. Drift times are indicated in bins (arbitrary units). Measurements were conducted in negative ion mode (i.e. on the [M–H]− species) in order to make sure that none of the gas-phase isoform arose from different sites of cationization on the oligosaccharide. Thus, the drift times cannot be directly compared to those measured in Fig. 2, neither the relative proportion of the two main populations of DP4 differentiated by their ion mobility for MLG and Lichenan. Figure S2. Ion mobility recorded in positive ionization mode for the oxidized DP4 species released from the incubation of PaLPMO9H with cellulose, MLG, Lichenan. A:Ketone species (m/z 687.2 Da) B: Gem-diol species (m/z 705.2 Da). Drift times are indicated in bins (arbitrary units). Figure S3. Ion mobility recorded in positive ionization mode for the non-modified DP5 species (m/z 851.2 Da) released from the incubation of PaLPMO9H with cellulose (black trace), MLG (green trace), Lichenan (red trace). Drift times are indicated in bins (arbitrary units). Figure S4. Analysis of the tetradecasaccharide XXXGXXXG substrate, prior incubation with the PaLPMO9H. A. ESI–MS spectrum showing the minor presence of XXLGXXXG or XXXGXXLG species. B. High-performance anion-exchange chromatogram indicating the presence of minor peaks likely corresponding to XXLGXXXG and XXXGXXLG. Peak integration reveals that they represent ca. 5% of the total oligosaccharide content. Figure S5. Tandem MS spectrum of the species displaying a +16 Da mass shift from the unmodified DP4 produced by PaLPMO9H from GM (detected at m/z 705.2). The observed fragments indica [file 13068_2017_749_MOESM1_ESM.docx]
